# Estimation of Fine Particulate Matter in Taipei Using Landuse Regression and Bayesian Maximum Entropy Methods

**DOI:** 10.3390/ijerph8062153

**Published:** 2011-06-14

**Authors:** Hwa-Lung Yu, Chih-Hsih Wang, Ming-Che Liu, Yi-Ming Kuo

**Affiliations:** 1 Department of Bioenvironmental Systems Engineering, National Taiwan University, Taipei 10617, Taiwan; E-Mails: hlyu@ntu.edu.tw (H.-L.Y.); r96622049@ntu.edu.tw (C.-H.W.); mingche_liu@mail.fcu.edu.tw (M.-C.L.); 2 Department of Design for Sustainable Environment, Ming Dao University, 369 Wen-Hua Rd., Peetow, Chang-Hua 52345, Taiwan

**Keywords:** Bayesian maximum entropy, landuse regression, particulate matter

## Abstract

Fine airborne particulate matter (PM_2.5_) has adverse effects on human health. Assessing the long-term effects of PM_2.5_ exposure on human health and ecology is often limited by a lack of reliable PM_2.5_ measurements. In Taipei, PM_2.5_ levels were not systematically measured until August, 2005. Due to the popularity of geographic information systems (GIS), the landuse regression method has been widely used in the spatial estimation of PM concentrations. This method accounts for the potential contributing factors of the local environment, such as traffic volume. Geostatistical methods, on other hand, account for the spatiotemporal dependence among the observations of ambient pollutants. This study assesses the performance of the landuse regression model for the spatiotemporal estimation of PM_2.5_ in the Taipei area. Specifically, this study integrates the landuse regression model with the geostatistical approach within the framework of the Bayesian maximum entropy (BME) method. The resulting epistemic framework can assimilate knowledge bases including: (a) empirical-based spatial trends of PM concentration based on landuse regression, (b) the spatio-temporal dependence among PM observation information, and (c) site-specific PM observations. The proposed approach performs the spatiotemporal estimation of PM_2.5_ levels in the Taipei area (Taiwan) from 2005–2007.

## 1. Introduction

Numerous studies over the last two decades indicate that the air quality measure of fine PM particles (PM_2.5_, particulate matter particles with an aerodynamic diameter ≤2.5 μm) can be more indicative of potential threats to human health than the commonly and long-used air quality measures of coarse particles, *i.e.*, PM_10_ (particulate matter particles with an aerodynamic diameter ≤10 μm) and total suspended particles (TSP). An increase in long-term exposure to PM_2.5_ is closely associated with increased mortality and diseases, such as lung cancer and cardiopulmonary disease [1–4]. Despite the long history of air quality monitoring throughout the entire island of Taiwan from 1983, and much like many other countries, its PM_2.5_ monitoring network did not begin to operate systematically and regularly until August 2005. The lack of long-term PM_2.5_ measurements prevents epidemiologists from assessing the chronic health effects of long-term exposure to PM_2.5_. Geostatistical techniques have been applied to estimate the spatiotemporal distributions of PM_2.5_ before the establishment of PM_2.5_ monitoring networks [5–8]. The ratio of PM_2.5_/PM_10_ is often used as an important indicator to characterize the underlying atmospheric processes within the local environment [7,8]. However, PM_2.5_/PM_10_ ratios can vary with time and space, depending on the landuse and emission patterns of the space-time location. For example, these ratios are approximately 0.69 and 0.52, respectively, in the urban and suburb areas of Shanghai (China) [9], about 0.45 among five different Asian regions (Australia, Hong Kong, Korea, Philippines, Vietnam, and Japan) [10], and range from 0.39 to 0.69 in urban and semi-rural areas of the United States [11]. Previous research provides a summary of PM_2.5_/PM_10_ ratios in megacities around the world [12]. Intra-urban ratios change significantly in Taipei, with a PM_2.5_/PM_10_ ratio of approximately 0.82 around the Bei-tou incinerator [13], 0.68 in high traffic areas, and 0.57 in downtown areas [14].

The spatial and temporal variation of PM_2.5_, PM_10_, and other air quality levels in Taiwan are generally high due to their high association with local emission patterns and meteorological conditions. Recent developments have been focusing on quantifying the levels of PM_2.5_, PM_10_, and other air quality observations using the surrogates of local emissions [15,16]. The landuse regression technique (LUR) has been widely applied to determine the linear relationship between air quality measures and landuse information and generate air quality maps with high spatial resolution [17–21]. In general, LUR air quality maps can delineate the significant contributions of certain geographical objects, such as highways. However, due to changes in meteorological conditions and limited landuse information, the quantitative results of air quality levels by LUR can vary from time to time. Therefore, the LUR is generally used to quantify the long-term average air quality levels in space [20–23]. Studies show that landuse information also plays an important role in the variation of the PM_2.5_/PM_10_ levels due to traffic and road emissions [24,25]. This is because the influence degree to PM_2.5_ and PM_10_ varies across different local landuse patterns. In addition, the temporal variations of PM_2.5_/PM_10_ resulting from the change of meteorological conditions can be less significant than the direct observations of PM_2.5_ and PM_10_. These characteristics make the PM_2.5_/PM_10_ ratio a proper surrogate of air quality patterns, which quantify the contributions of spatial variations in landuse patterns. However, relatively few studies investigate the relationship between the PM_2.5_/PM_10_ ratios and landuse information.

This study investigates the spatiotemporal distribution of PM_2.5_ across the Taipei area from 2005–2007 by integrating the information of PM_10_ and landuse information. This study uses LUR to establish a quantitative relationship between PM_2.5_/PM_10_ and landuse information. The Bayesian maximum entropy (BME) method is then used to assimilate the PM_2.5_ and the secondary information from the LUR analysis. The comparison is made by assessing the improvement of PM_2.5_ prediction accuracy with the incorporation of the secondary information, *i.e.*, geostatistical estimation by (1) only PM_2.5_, (2) both PM_2.5_ and PM_10_ and (3) PM_2.5_, PM_10_ and landuse information.

## 2. Materials

### 2.1. Study Area

Taipei, including Taipei city and Taipei county, is the largest metropolitan area in Taiwan, and has a vehicle density as high as 6,000 vehicles per km^2^. In addition to traffic emissions, three incineration plants are major sources of pollutants in the area [26].

**Figure 1.**
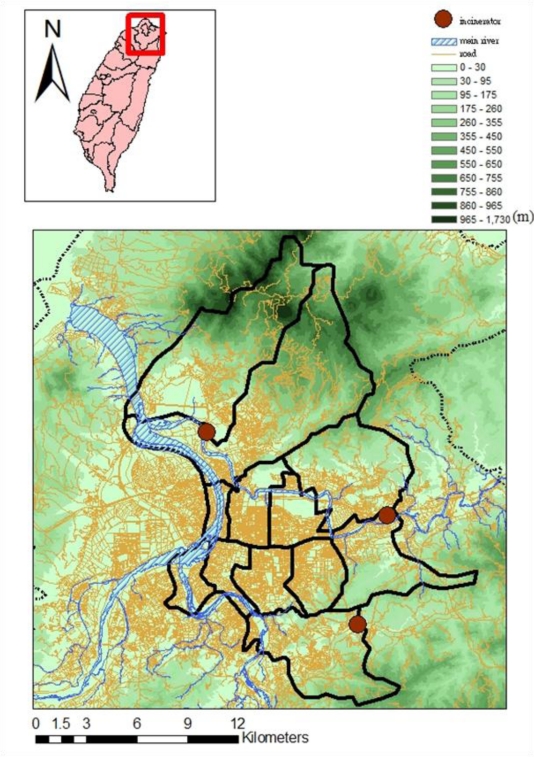
The highways, rivers, and topography in the Taipei metropolitan area.

The Taipei area is bounded by mountains, *i.e.*, Yangming Mountains to the north, Linkou mesa to the west, and a ridge of the Snow Mountains to the southeast. These mountains form the second largest basin of the island (Figure 1). This basin topography increases the concentration level of ambient pollutants and creates a high contrast between the urbanization of the basin floor in Taipei and the surrounding mountain areas.

### 2.2. Ambient Pollutant Data

An island-wide monitoring network operated by Taiwan Environmental Protection Agency (TWEPA) regularly records ambient pollutants, *i.e.*, criteria pollutants such as PM, ozone, NO_x_, CO, SO_2_ [27], and meteorological variables. There are 18 TWEPA stations within the Taipei metropolitan area, and these stations recorded both PM_2.5_ and PM_10_ from 2005–2007. Table 1 summarizes the PM_2.5_ and PM_10_ statistics.

**Figure 2.**
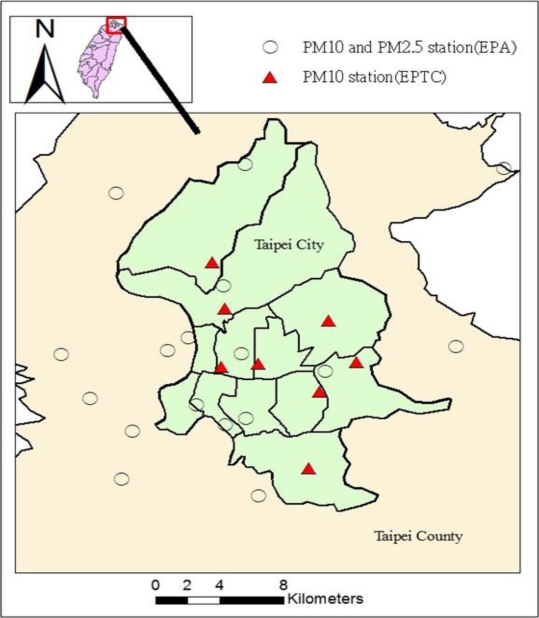
Spatial distribution of PM_10_ and PM_2.5_ monitoring stations in Taipei.

In addition, the Department of Environmental Protection and the local governments of Taipei city and Taipei county (TPEDEP) have independently collected PM data since 1970 and 1990, respectively. However, only the Taipei city government records PM_10_ on a daily basis at its eight stations. This study uses the PM_2.5_ and PM_10_ data from both central and local governments to estimate the monthly PM_2.5_/PM_10_ ratios at every PM station (Figure 2). This study aggregates the PM_2.5_ and PM_10_ data into monthly data following the procedure suggested by USEPA [28]. The monthly PM_2.5_ levels at the TPEDEP stations were estimated by the BME method as discussed below with only PM_2.5_ observations. The estimated monthly PM_2.5_ and PM_10_ were then used to obtain the spatiotemporal distribution of PM_2.5_/PM_10_ ratios for all stations from 2005–2007. The other observed ambient pollutants, *i.e.*, CO, NO_2_, SO_2_, and O_3_, were used as the emission indicators, as discussed below.

### 2.3. Landuse Data

The National Land Surveying and Mapping Center (Taiwan) conducted a comprehensive landuse surveying of the entire Taipei area in 2007. This survey includes nine major classes of land usage, including agriculture, forest, traffic, water, buildings, utilities, recreation areas, mining areas, and others, *i.e.*, transportation data discussed below. Each of the major landuse categories mentioned above includes more detailed classifications [29] This study analyzes the potential major or minor landuse classes that may have positive or negative effects on the air quality levels. The selection criteria include significant variables identified in previous studies, e.g., roads, and insights from local experts [30], e.g., motorcycles. The selected landuse classes include the areas of farms, forests, railroad, freeway, highway, roads, ports, government institutions, school, commerce, residence, industry, hospital, social welfare facilities, public utilities, and parks.

**Figure 3.**
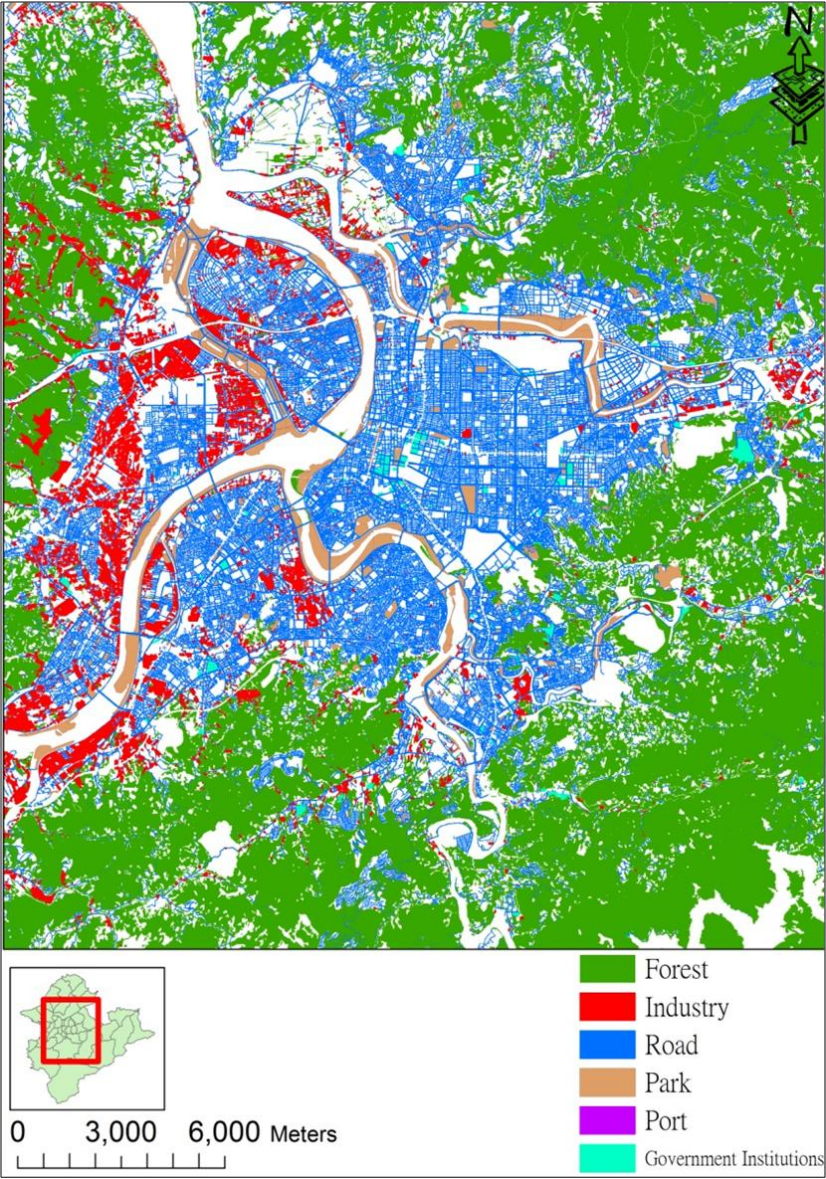
Spatial distribution of landuse patterns in Taipei area.

Figure 3 shows the spatial distribution of some landuse classes in Taipei. This figure clearly shows that city development is concentrated in the plains of the Taipei basin floor. In addition, this study generates spatiotemporal traffic information by uniformly assigning the recorded number of various registered vehicles [31,32] to the study area based on the road areas identified by the landuse data. The vehicle types of this analysis include motorcycle, bus, passenger car, and truck.

## 3. Methods

This study uses a landuse regression method to determine the relationship between PM_2.5_/PM_10_ and local emission-related information. The emission-related information in this study includes non-PM ambient pollutants and landuse data. Local emission-related data are derived by GIS functions which estimate this size or area of selected indicators within the specified spatial buffers. Various spatial ranges of buffers are used for landuse information surrounding the PM_2.5_/PM_10_ data (*i.e.*, 0–50 m, 50–100 m, 100–300 m, 300–500 m, and 500–1,000 m) to determine the different ranges of transport processes produced by different types of emissions. The relationship between the sizes/proximity of local emission-related data and PM_2.5_/PM_10_ ratios is assumed to be homogeneous over the entire study area, and can therefore be formulated in a linear form. Multivariate stepwise regression analysis was performed to select the most significant regressors and estimate their associated parameters. Due to the high linear dependencies among the selected emission-related variables in the landuse regression model, this study uses the variance inflation factor (VIF) to identify multicolinearity among the regressors and avoid potentially dubious results from the analysis [33]. This study uses SPSS software for landuse regression analysis.

The BME method mathematically represents air pollution attributes (*i.e.*, PM measurements and ratios) in terms of spatiotemporal random fields (S/TRF; [34]). Let *X***_p_** = *X***_s_**_,_*_t_* denote a S/TRF of an air pollution attribute, where the vector **p** = (**s**,*t*)denotes a spatiotemporal point (**s** is the geographical location and *t* is the time). The S/TRF model is a collection of all physically possible realizations of the attribute to be represented mathematically. The S/TRF model is fully characterized by its probability density function (pdf), *f_KB_*, where the subscript KB denotes the ‘knowledge base’ used to construct the pdf. In particular, BME considers a distinction between: (a) the general KB, denoted by G-KB, and (b) the site-specific KB, S-KB. The total KB is denoted as *K* = *G* ∪ *S*, *i.e.*, it includes both the general and the site-specific KB. The fundamental BME equations are as follows (for technical details, see [35,36]):

(1) $$\begin{array}{l} \;\;\;\;\;\;\;\;\;\;\int d\chi (\text{g}-\bar{\text{g}})e^{\mu^{T}\text{g}}=0\\ \int d\chi \xi_{S}e^{\mu^{T}\text{g}}-Af_{K}(\chi )=0 \end{array}\},$$

where ***g*** is a vector of *g_α_* -functions (*α* = 1,2,...) that stochastically represents the G-KB under consideration (the bar denotes statistical expectation), ***μ*** is a vector of *μ_α_* -coefficients that depends on the space-time coordinates and is associated with ***g*** (*i.e.*, *μ_α_* expresses the relative significance of each *g_α_* -function in the composite solution sought), *ξ_S_* represents the S-KB available, *A* is a normalization parameter, and *f_K_* is the pollutant pdf at each space-time point (the subscript K means that *f_K_* is based on the blending of the core and site-specific KB). The terms ***g*** and *ξ_S_* the inputs in Equation (1), whereas the unknowns are the ***μ*** and *f_K_* across space-time.

The G-KB refers to the entire ***p*** -domain of interest, which consists of the space-time point vector ***p****_k_*, where attribute estimates are sought, and the point vector ***p***_data_, where site-specific information is available. The G-KB may include theoretical space-time dependence models (mean, covariance, variogram, generalized covariance, multiple-point statistics, and continuity orders) of the air pollution attribute *X***_p_** [37,38]. The *S* -KB includes physical data ***χ****_data_* obtained at points ***p****_i_* (*i* = 1,2,…, *m*) of the specified geographical area, *i.e.*, the various kinds of PM measurements or ratios are considered part of the *S* -KB and are expressed by *S* :***χ***_data_ = (***χ***_hard_, ***χ***_soft_) = (*χ*_1_,…, *χ_m_*) where the ***χ***_hard_ = (*χ*_1_,…, *χ*_*m*_*h*__) denote hard data at points ***p****_i_* (*i* = 1,2,…, *m_h_*) that are exact PM measurements (*i.e.*, the hard ***χ***_hard_ occur with probability one); and the ***χ***_soft_ = (*χ*_*m*_*h*_+1_,.…, *χ_m_*) denote soft data at points ***p****_i_* (*i* = *m_h_* +1,…, *m*) that may include uncertain evidence and secondary information. This study represents the soft PM data from landuse regression model in the interval, *I_S_*, *i.e.*, ***χ***_soft_ : {*χ_i_* ∈ *I_i_* =[*l_i_*, *u_i_*],*i* = *m_h_* + 1,…,*m*}; For other examples of soft data, see [39,40].

This study characterizes both PM_2.5_, *X_s_*_,_*_t_* and PM_10_, *Y_s_*_,_*_t_*, concentrations by S/TRF. The PM_2.5_/PM_10_ ratio can be represented as *r_s_*_,_*_t_* = *X_s_*_,_*_t_*/*Y_s_*_,_*_t_*, where *t* is the time in months during the period 2005–2007. The values of *r_s_*_,_*_t_*, can be estimated monthly at each PM monitor station based on the recorded or estimated PM data. The monthly *r_s_*_,_*_t_*, of PM_2.5_/PM_10_ ratios were calculated at the monitoring stations where daily PM_2.5_ and PM_10_ data are both available and the eight PM_10_ stations operated by TPEDEP where only daily PM_10_ data was observed. During the study period, the *r***_p_** -values at the stations were assumed to be an empirical function of the emission-related indicators by LUR. The spatiotemporal distribution of the ratios across the study area is estimated based on this empirical relationship and the citywide emission information obtained using ArcGIS 9.2. Note that the uncertainty is prevalent in the estimation of spatiotemporal distribution of the ratios. The ratios at each space-time location are assumed to be uniform-distributed with intervals of ⌊*r̂_s_*_,_*_t_* – *SD*(*r̂_s_*_,_*_t_*), *r̂_s_*_,_*_t_* + *SD*(*r̂_s_*_,_*_t_*)⌋, where *r̂_s_*_,_*_t_* and *SD*(*r̂_s_*_,_*_t_*) represent the ratio estimation and its standard deviation from LUR, respectively. The multiplication of PM_2.5_/PM_10_ ratios and PM_10_ generates an uncertain spatiotemporal trend of PM_2.5_. To account for the uncertainty in the ratio estimation and subsequent trend estimation in space and time, this study uses the BME method for the spatiotemporal estimation of the PM_2.5_ with the uniform-distributed PM_2.5_ residuals which upper and lower bounds are derived from the intervals of trend estimations and the PM_2.5_ observations. In summary, this study applies the two-stage approach to integrate landuse regression and BME methods for spatiotemporal PM_2.5_ estimations, in which landuse regression is used to characterize the spatial variability of PM_2.5_, *i.e.*, ratios, and BME performs later by assimilating the uncertainty by landuse regression and the spatiotemporal dependence for the modeling of spatiotemporal PM_2.5_ distribution.

## 4. Results

Table 2 lists the selected variables from the emission-related dataset in LUR model by the stepwise regression method. This table lists variables by the rank of their significance to the variation of PM_2.5_/PM_10_ ratios. Most of the selected variables can elevate the level of PM_2.5_/PM_10_ ratios. The road, forest, industrial area, and park landuse patterns has the greatest effect on increasing the PM_2.5_/PM_10_ ratios. Most selected ranges of the variables are 500 m–1,000 m.

This implies that the level of PM_2.5_/PM_10_ represents the general air quality patterns of the area surrounding the monitoring stations rather than the direct emission impact from the short distances. The only selected variable that shows the ability to reduce PM_2.5_/PM_10_ values is the park landuse pattern, which ranges between 300 m and 500 m. Note that most traffic information is not included in the model due to multicollinearity with the spatial distribution of road area. The exception is the bus volume, which can increase the local ratio level within.

**Figure 4.**
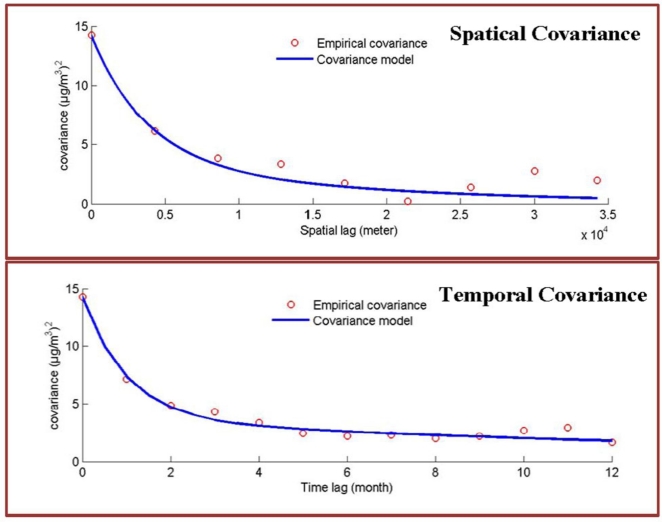
Spatiotemporal covariance of PM_2.5_ (top) pure spatial covariance (bottom) pure temporal covariance.

The spatiotemporal distribution of monthly PM_2.5_ can be obtained by multiplying the empirical functional of landuse information, *i.e.*, the LUR model and PM_10_ variation in space and time. However, the spatiotemporal dependence among the PM_2.5_ is not considered. This study integrates the BME method with LUR to model the high frequency part of the PM_2.5_ variation in space and time, *i.e.*, the unexplained PM_2.5_ noise in the LUR model. The high frequency part of spatiotemporal variation of PM_2.5_ is characterized by the stationary nested covariance shown below (see Figure 4):

(2) $$c(h,\tau )=c_{0}\text{exp}(-\frac{3h}{a_{r1}})\text{exp}(-\frac{3\tau }{a_{t1}})+c_{1}\text{exp}(-\frac{3h}{a_{r2}})\text{exp}(-\frac{3\tau }{a_{t2}})$$

where [*c*_0_,*c*_1_]=[10.5, 3.729], [*a_r_*_1_,*a_r_*_2_]=[11.092 km, 50 km] and [*a_t_*_1_,*a_t_*_2_]=[3 month, 50 month]. The BME method integrates the probabilistic data of PM_2.5_ residuals and spatiotemporal covariance model in Equation (2) to generate the monthly spatiotemporal distributions of PM_2.5_ from 2005–2007.

**Figure 5.**
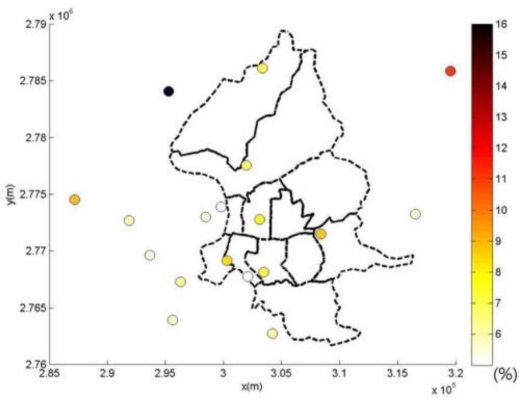
Spatial distribution of relative error of PM_2.5_ estimations by LUR model.

**Figure 6.**
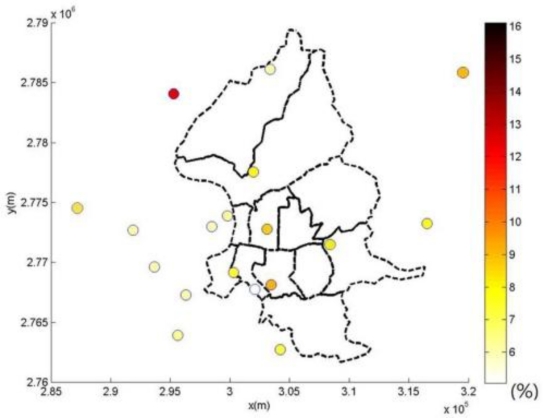
Spatial distribution of relative error of PM_2.5_ estimations by the integration of LUR and BME methods.

This study compares the modeling of spatiotemporal PM_2.5_ distribution using the kriging method, LUR method, and the integration of LUR and BME methods, respectively (Table 3). The kriging estimation is based upon the modeling of PM_2.5_ observations directly, and ignores their uncertainty [35]. Leave-one cross-validation results show that the LUR model outperforms the kriging method in PM_2.5_ estimations. Furthermore, the BME method can improve the accuracy of PM_2.5_ estimation in this study. Figure 5 and Figure 6 show the spatial distribution of estimation performance at each PM_2.5_ observation location by the LUR model and BME method, respectively.

**Figure 7.**
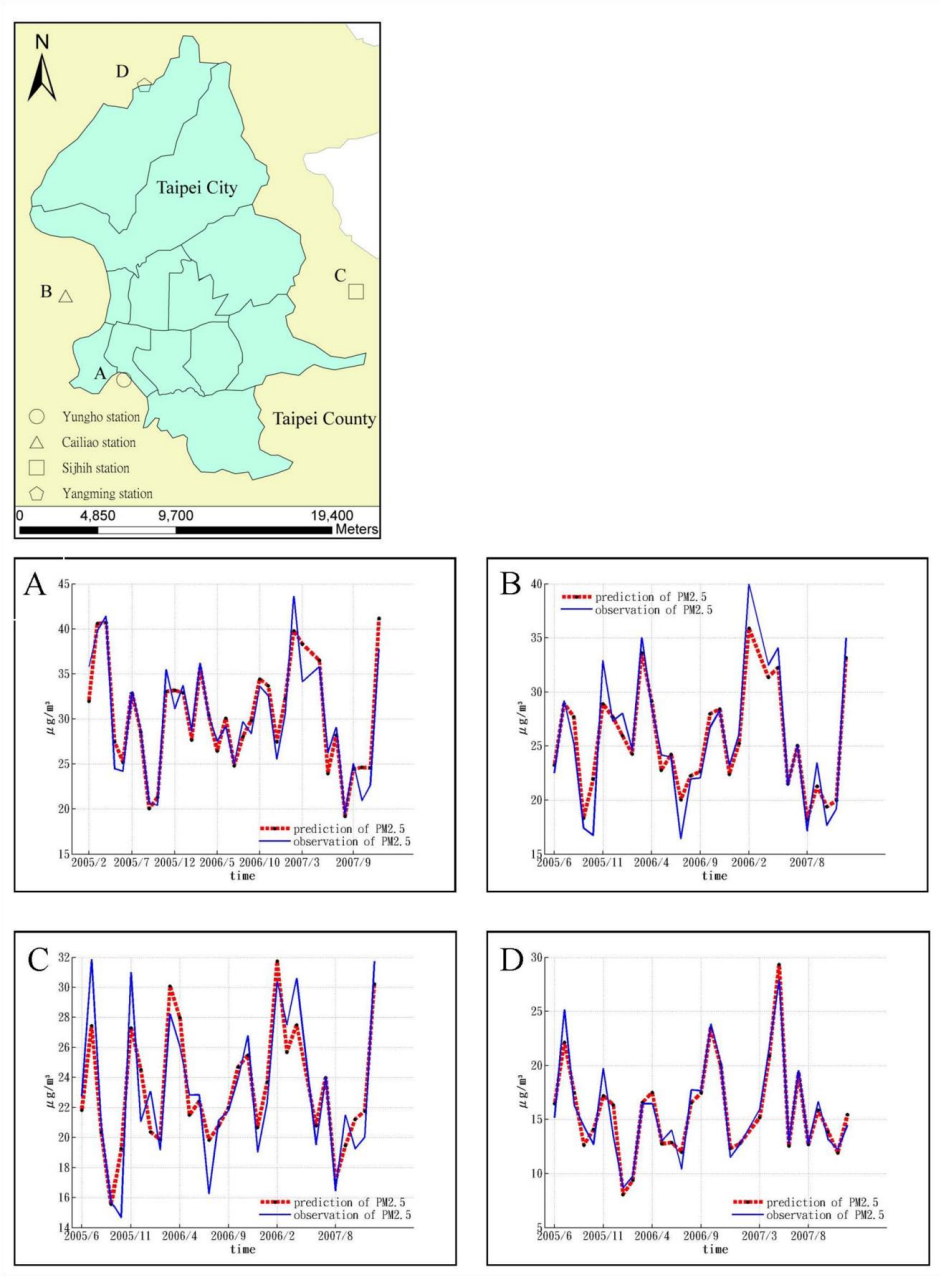
A comparison of PM_2.5_ observations and estimations at four PM_2.5_ stations: **(A)** Yungho station, **(B)** Cailiao station, **(C)** Sijhih station, and **(D)** Yangming station.

Figure 7 shows the temporal variation of monthly PM_2.5_ observations and their estimations by BME method at the four selected locations, *i.e.*, Yungho, Cailiao, Sijhih, and Yangming. The selected locations represent different parts of Taipei area.

## 5. Discussion

This study uses the BME method to integrate the LUR model in the prediction (estimation) of fine particulate matter concentrations across space-time in the Taipei metropolitan area. This implementation of BME theory allows this study to determine attribute distributions in a composite space-time domain without restrictive or unrealistic assumptions (such as linearity, normality, independency *etc.*). The general knowledge base of the BME method used to characterize the general pattern of PM_2.5_ is based on the empirical relationship between landuse information and the LUR model. Many studies [18,20,41] show that the LUR is able to produce high-resolution air quality maps and address the effects of each landuse pattern. However, updating a landuse database often requires tremendous efforts, making it difficult to update the information of landuse changes over time. To characterize the general pattern of PM_2.5_ in space and time, the emission-related database in this study includes the variables of non-PM ambient pollutants and traffic information, which change over time and are considered to be highly associated with the level of PM_2.5_. As a useful indicator of local emission patterns [7,8], this study determines PM_2.5_/PM_10_ ratios based on landuse distribution and some LUR model emission information mentioned above. As expected, most of the significant variables in the LUR model of ratios are pure spatial information, *i.e.*, certain landuse patterns within the certain distances from the observation locations. This implies that the spatial variation of PM_2.5_/PM_10_ ratios exceeds its temporal variation, *i.e.*, the effects of landuse data to PM_2.5_/PM_10_ ratios in Taipei are more important than other temporal factors, such as meteorological and seasonal effects. In addition, the monthly ratios mostly characterize the general emission pattern. Therefore, the ranges with greatest size of area at each neighborhood appear most frequently in this study. The factors included in this study are mostly variables which can increase the level of ratios. Some of the potential variable can significant reduce the level of ratios are selected, e.g., forest and park. Among them, the contradiction of the park effect from different ranges may be due to the common spatial distribution of the urban setting in Taipei, in which major parks are commonly located near high-density urbanized areas. Thus, only locations immediately next to parks can enjoy have the advantages of the park’s ability to improve air quality. As for areas situated further from the parks, the air quality levels can easily be elevated by other contributing factors. This is partially responsible for the high variability of PM_2.5_ levels, which can increase significantly based on local emissions and decrease significantly when emission sources are removed.

Covariance analysis shows that the PM_2.5_ exhibits two spatiotemporal interactions with different space-time ranges. These interactions represent the local and long-term transport patterns of fine particulate matter over the Taipei area with the two distinct space-time ranges: [11 km, 3 months] and [50 km, 50 months]. The dominant process of PM_2.5_ distribution is the local transport with spatial and temporal extents of 11 km and 3 months. The spatial extent considers the size of highly-urbanized areas, while the temporal range shows how seasonal effects play an important role in the concentration level of PM_2.5_. The variability of long-term process can result from the mass dispersion over the continents, such as dust storms, due to certain meteorological conditions [42–45].

Figure 4 and Figure 5 show the spatial distributions of performance assessment for the LUR and BME methods. Results show that both analyses obtain similar spatial patterns of accuracy distribution, and can perform relatively better in areas with better PM observations. The analysis of the LUR model assumes a homogenous relationship between landuse information and PM observations across space and time. However, the heterogeneity of the statistical relationship between the landuse and PM concentration may vary from location to location due to distinct causality between analysis attributes. This spatial unbalance of information support causes the homogeneous relationship address the area of abundant information better. This results in distinct performance differences between the central and boundary areas in spatial distribution of cross-validation results of the LUR and BME methods, especially in Figure 4. Though the BME method shares the same spatiotemporal patterns as the LUR model, the inclusion of spatiotemporal dependence in the BME method reduces the effects of unbalance information and improves the estimation accuracy, as Figure 5 shows.

Table 3 shows the advantages of integrating landuse information in spatiotemporal estimation in PM_2.5_. Cross-validation comparison shows that the LUR model offers greater improvement than the kriging method, *i.e.*, the most-widely used geostatistical method. The LUR and kriging methods only consider landuse information and spatiotempral dependence among PM_2.5_, respectively. Table 3 shows that the BME method achieves the smallest mean square error, standard deviation, and other statistics in PM_2.5_ estimation errors. Figure 6 compares the temporal distribution of PM_2.5_ observations and BME estimations for four selected locations. The four locations were selected to represent the East, South, West, and North parts of the city, respectively. Results show that, for all locations, the BME estimations generally achieved good agreement with the PM_2.5_ observations.

## 6. Conclusions

This study discusses the application of spatiotemporal statistics to science-based PM_2.5_ mapping in Taipei. The main goals of the BME method are to generate PM_2.5_ maps in a composite space-time domain, in which the core knowledge in the form of empirical laws by LUR model with the informative secondary information derived from landuse data. Results show that incorporation of multi-sourced soft and hard information through BME analysis and mapping can effectively improve the accuracy of PM_2.5_ estimation across space-time. This analysis demonstrates the most influential landuse patterns elevating PM_2.5_ levels. In addition, the two dominant space-time mechanisms underlying PM_2.5_ space-time distributions in Taipei include local and long-term transport processes.

## Figures and Tables

**Table 1.** Summary of statistics of hourly PM_10_ and PM_2.5_ observations from 2005–2007 (unit: μg/m^3^).

**Pollutants**	**Average**	**Standard deviation**	**Median**	**Minimum**	**Maximum**
PM_2.5_	28.92	8.48	28.29	9.31	81.60
PM_10_	54.24	33.26	47.04	0.83	598.25

**Table 2.** Coefficients of selected variables of LUR model.

**Variable (m^2^)**	**Spatial Buffer (meters)**	**Coefficient (10^−7^)**
Road	500–1,000	6.608
Forest	500–1,000	2.552
Industry	300–500	33.11
Park	500–1,000	8.745
Railroad	0–50	10,000
Government institutions	100–300	117.2
Park	300–500	−21.13
Public Equipment	100–300	493.3
Bus	0–50	20,000
Public Equipment	0–50	815.4
Port	500–1,000	48.45

**Table 3.** Results of cross validation.

**Method**	**Mean error**	**Standard Deviation**	**Median**	**Max value of error**	**Min value of error**
Landuse + BME	2.1560	2.0584	0.0889	8.4393	−15.390
Landuse	2.7865	2.5685	−0.1035	10.8316	−16.6528
kriging	3.1816	2.7798	−0.006	14.3380	−15.7980
